# Urinary metabolomics reveals kynurenine pathway perturbation in newborns with transposition of great arteries after surgical repair

**DOI:** 10.1007/s11306-019-1605-3

**Published:** 2019-10-28

**Authors:** Manuela Simonato, Igor Fochi, Luca Vedovelli, Sonia Giambelluca, Cristiana Carollo, Massimo Padalino, Virgilio P. Carnielli, Paola Cogo

**Affiliations:** 1 Anesthesiology and Intensive Care Unit, Department of Medicine-DIMED, University of Padova, Padua, Italy; 2 PCare Laboratory, Fondazione Istituto di Ricerca Pediatrica, “Citta’ della Speranza”, Padua, Italy; 3 Thermo Fisher Scientific, Milan, Italy; 4 Women and Child Health Department, University of Padova, Padua, Italy; 5 Pediatric and Congenital Cardiac Surgical Unit, Department of Cardiac, Thoracic and Vascular Sciences, Padova University, Padua, Italy; 6 Division of Neonatology, Department of Clinical Sciences, Polytechnic University of Marche and Azienda-Ospedaliero Universitaria Ospedali Riuniti, Ancona, Italy; 7 Division of Pediatrics, Department of Medicine, Udine University, Udine, Italy

**Keywords:** Untarget metabolomics, Transposition of great arteries, Kynurenine pathway, UHPLC-HRMS, Newborns

## Abstract

**Introduction:**

Transposition of the great arteries (TGA) is a cyanotic congenital heart defect that requires surgical correction, with the use of cardiopulmonary-bypass (CPB), usually within 3 weeks of life. The use of CPB in open heart surgery results in brain hypoperfusion and in a powerful systemic inflammatory response and oxidative stress.

**Objective:**

We aimed to develop a novel untargeted metabolomics approach to detect early postoperative changes in metabolic profile following neonatal cardiac surgery.

**Methods:**

We studied 14 TGA newborns with intact ventricular septum undergoing arterial switch operation with the use of CPB. Urine samples were collected preoperatively and at the end of the surgery and were analyzed using an untargeted metabolomics approach based on UHPLC-high resolution mass spectrometry.

**Results:**

Since post surgery metabolic spectra were heavily contaminated by metabolites derived from administered drugs, we constructed a list of drugs used during surgery and their related metabolites retrieved from urine samples. This library was applied to our samples and 1255 drugs and drug metabolites were excluded from the analysis. Afterward, we detected over 39,000 unique compounds and 371 putatively annotated metabolites were different between pre and post-surgery samples. Among these metabolites, 13 were correctly annotated or identified. Metabolites linked to kynurenine pathway of tryptophan degradation displayed the highest fold change.

**Conclusions:**

This is the first report on metabolic response to cardiac surgery in TGA newborns. We developed an experimental design that allowed the identification of perturbed metabolic pathways and potential biomarkers of brain damage, limiting drugs interference in the analysis.

**Electronic supplementary material:**

The online version of this article (10.1007/s11306-019-1605-3) contains supplementary material, which is available to authorized users.

## Introduction

D-loop Transposition of the Great Arteries (D-TGA) is the second most common form of cyanotic congenital heart disease (CHD). TGA accounts for 5% to 7% of all CHD, with a birth prevalence of 0.31 per 1000 live births (95% CI 0.28–0.34) and male preponderance (van der Linde et al. 2011). The anatomical defect of D-TGA leads to cyanotic heart disease because of two parallel circulations. The first one sends deoxygenated systemic venous blood to the right atrium and back to the systemic circulation via the right ventricle and aorta; the second one sends oxygenated pulmonary venous blood to the left atrium and back to the lungs via the left ventricle and pulmonary artery. This will lead to profound hypoxic state pre- and postnatally until cardiac surgery is accomplished.

Survival without surgical repair is different among subset. It is very poor in untreated patients with D-TGA and intact ventricular septum: 80% at 1 week but only 17% at 2 months and 4% at 1 year (Mitchell et al. 1971). Neonatal arterial switch operation (ASO) is the treatment of choice for infants with D-TGA and it is usually performed within 3 weeks of life (Authors/Task Force et al. 2017).

Survival into adulthood after ASO is common, with a 20-year survival rate of nearly 90% (Villafane et al. 2014). However, early- and late-onset neurodevelopment (ND) abnormalities commonly occur. Although there has been a decline in gross neurological insults in D-TGA patients, many experienced behaviour, speech, and language delays at 4 and 8 years, with significant deficits in visual-spatial and memory skills as well as in components of executive functioning such as working memory and sustained attention (Bellinger et al. 2003). Emerging evidence has shown a correlation between perioperative events such as significant hypoxemia, acidosis, prolonged hypothermia during cardiopulmonary-bypass (CPB) and ND and behavioral testing (Vedovelli et al. 2018).

In the peri- and post-operative phases, some centers use near infrared spectroscopy, transcranial Doppler, and measurement of biomarkers such as S100β and GFAP to identify infants at higher risk for ND abnormalities (Andropoulos et al. 2010). Despite their clear utility, the measured biomarker level provides only a partial snapshot of an individual’s current physiological state. While this provides useful information, single measurements do not give details about molecular pathway perturbations underpinning cardiac surgery-induced brain damage.

Metabolomics is a powerful approach to facilitate the detection of temporary physiological changes in real-time and allows its use as a monitoring approach for the potential surgery insult. Since many hundreds of small molecules are measured in parallel, a metabolomics experiment provides an individual *metabolic profile*, or *fingerprint*, for each analysed sample. Analyzing metabolic differences between unperturbed and perturbed pathways could provide insight into underlying disease pathology and disease prognosis and diagnosis.

To the best of our knowledge, no studies have been published on the application of untargeted metabolomics to newborns with TGA undergoing cardiac surgery with CPB yet. Furthermore, relatively little is known about the metabolic changes that occur after cardiac surgery. A pilot study in children with different types of CHD found a difference in the metabolic profile of pre- and post-surgery plasma samples that returned toward baseline 48 h post-operatively (Correia et al. 2015). More recently, Davidson et al. (2018) used a targeted metabolomic approach to identify individual metabolites associated with post-operative complications in infants with less than 4 months undergoing cardiac surgery with CPB.

The metabolome is the final downstream product of gene transcription and protein translation; it changes rapidly, in timescales of minute to hours, and closely defines the phenotype. Because the dynamics of the human body are mirrored in the metabolome, we hypothesize that CPB-induced neuroinflammation/oxidative damage could be associated with an altered urine metabolite composition.To address this hypothesis, we performed high resolution mass spectrometry-based untargeted metabolomic of urine samples collected before and after CPB from newborns with TGA and we developed a novel data analysis workflow useful to clear the metabolites deriving from administered drugs out of the urinary metabolic profile. The application of this workflow resulted in the: (i) identification of key biochemical pathways perturbed during CPB, and (ii) selection of potential new biomarkers that can be used to discriminate the newborns at higher risk of worse post-operative outcome.

The ability to identify those TGA newborns at greater risk of long-term disability would provide personalized neuroprotective and therapeutic strategies, and hence improve long-term outcome.

## Methods

### Patients

The study was approved by the institutional review board and by the ethics committee of the Padova University Hospital. Fourteen newborns undergoing ASO for TGA correction were recruited pre-operatively, with informed parental consent. The study was conducted at the Pediatric Cardiovascular Surgery Unit, Centro ‘V. Gallucci’, Padova University Hospital, Italy.

Exclusion criteria were age greater than 3 weeks, adjusted gestational age < 37 weeks, previous heart surgery, hemodynamic instability, respiratory failure, factor V less than 20%, creatinine clearance less than 30%, confirmed or suspected errors of metabolism or chromosomal, and major neurological abnormalities before surgery.

### Patient biological samples

Urine samples were obtained from the urimeter of the Foley catheter system pre-operatively (at induction of anesthesia and before first surgical incision) and at the end of the surgery before initiation of modified ultrafiltration. Urine samples were centrifuged to remove cells and other debris. The supernatant was aliquoted and stored at − 80  °C.

### Sample processing

A “dilute-and-shoot” strategy was applied to the urine samples in order to minimize losses during sample preparation and because it is low time-consuming. Urine samples were thawed and centrifuged at 15,000×*g* for 20 min at 4  °C. Supernatant was diluted 1:5 and 1:2 with HPLC-grade water for positive (ESI+) and negative (ESI−) ionization analysis, respectively. Quality control (QC) samples were prepared by mixing equal volumes of all the urine samples. These pooled QC samples were prepared as described for real samples.

A procedural blank, used to monitor contamination acquired during all stages of sample preparation, a pool of the pre-surgery samples (pool-pre) and a pool of the post surgery samples (pool-post) were also prepared.

### Chemicals and reagents

LC–MS grade methanol, water and formic acid as well as the standard mixtures used for the external calibration of the MS instrument were from Thermo Fisher Scientific (MA, USA). All analytical grade reference compound (glycocholic acid, d-tryptophan, N-acetyl-l-tryptophan, L-kynurenine and Kynurenic acid) were from Sigma Aldrich (Steinheim, Germany).

### Liquid chromatography

The liquid chromatographic separation was done on a Thermo Scientific Dionex UltiMate 3000 RS system with a Hypersil Gold C-18 (100 × 2.1 mm, 3.0 μm, Thermo Fisher Scientific) column. The column was eluted at a flow rate of 300 µl/min with 80% of mobile phase A (0.02% formic acid in water) for 0.1 min followed by a linear gradient to 100% of mobile phase B (0.02% formic acid in methanol) over 20 min, then kept constant for 5 min, brought back to the initial conditions in 0.5 min, and maintained for 4 min. The column compartment and autosampler temperatures were set at 35  °C and 10 °C, respectively. The injection volume was 5 µl. Samples were randomly injected in triplicate to prevent any spurious classification deriving from the position of the sample in the sequence.

### Mass spectrometry

A Q Exactive Classic mass spectrometer equipped with heated-ESI-II (HESI-II) ion source was employed (Thermo Fisher Scientific). Two analytical sequences (one in ESI+ and one in ESI- ionization mode) were executed. The capillary temperature was set at 300 °C. Nitrogen sheath gas was set at a flow rate of 45 arbitrary units (AU). Nitrogen auxiliary gas was set at a flow rate of 12 AU. The positive and negative spray voltage were 3.3 kV and 2.7 kV, respectively. Two acquisition modes were used with an m/z range 100–1200. A full MS scan at 70,000 resolution, an auto gain control target under 5 × 10^5^ and a maximum injection time of 200 ms. The other acquisition mode was a full MS followed by data-dependent MS^2^ with a resolution of 17,500, an auto gain control target of 2 × 10^5^, a maximum injection time of 50 ms, a loop count of top 5 peaks and an isolation window of m/z 1.0. All MS^2^ spectra of the compounds were acquired at two collision energies, 30 and 60 eV. XCalibur ™ 4.1 software (Thermo Fisher Scientific) was used for data acquisition.

### Generation of the drug metabolites list

**Fig. 1:**
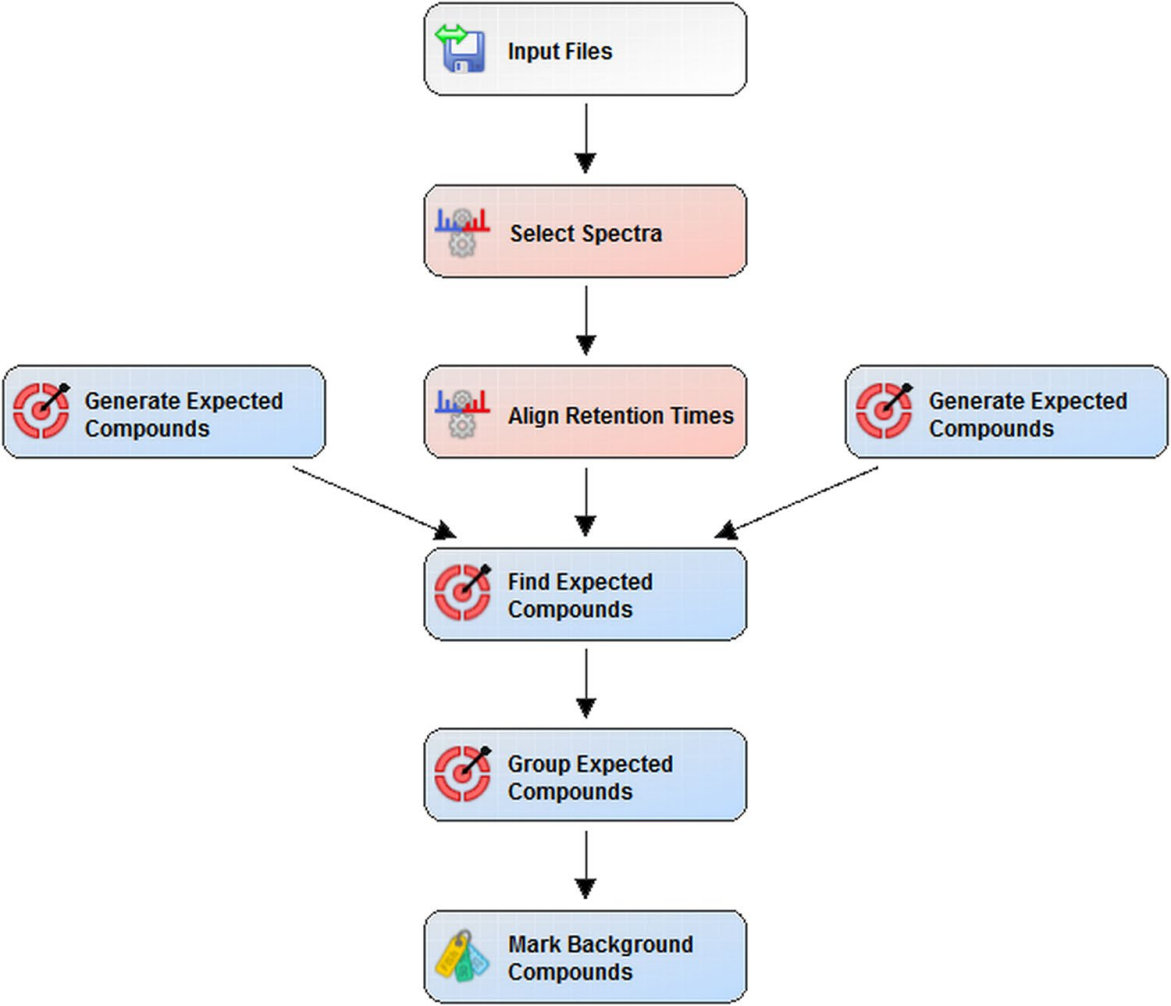
Workflow tree from the Compound Discoverer 2.1 software displaying select data processing nodes and the associated workflow connections. Preliminary data processing nodes included are: Imput Files, Select Spectra and Align Retention Times nodes. The “Generate Expected Compounds” node is used to generate a list of m/z values for the ionized compounds, in our case a drugs, that is expected to be in a sample. The list includes the parent compound and its possible transformation products. The “Find Expected Compound” node is used to search for compounds in the raw file using the ion list generated by the “Generate Expected Compounds” nodes. The “Group Expected Compounds” node combines chromatographic peaks across the raw files by using their molecular weight and retention time to group similar compounds. Lastly, the Mark Background Compounds node incorporates a procedural blank to indicate compounds arising from sample preparation

**Fig. 2:**
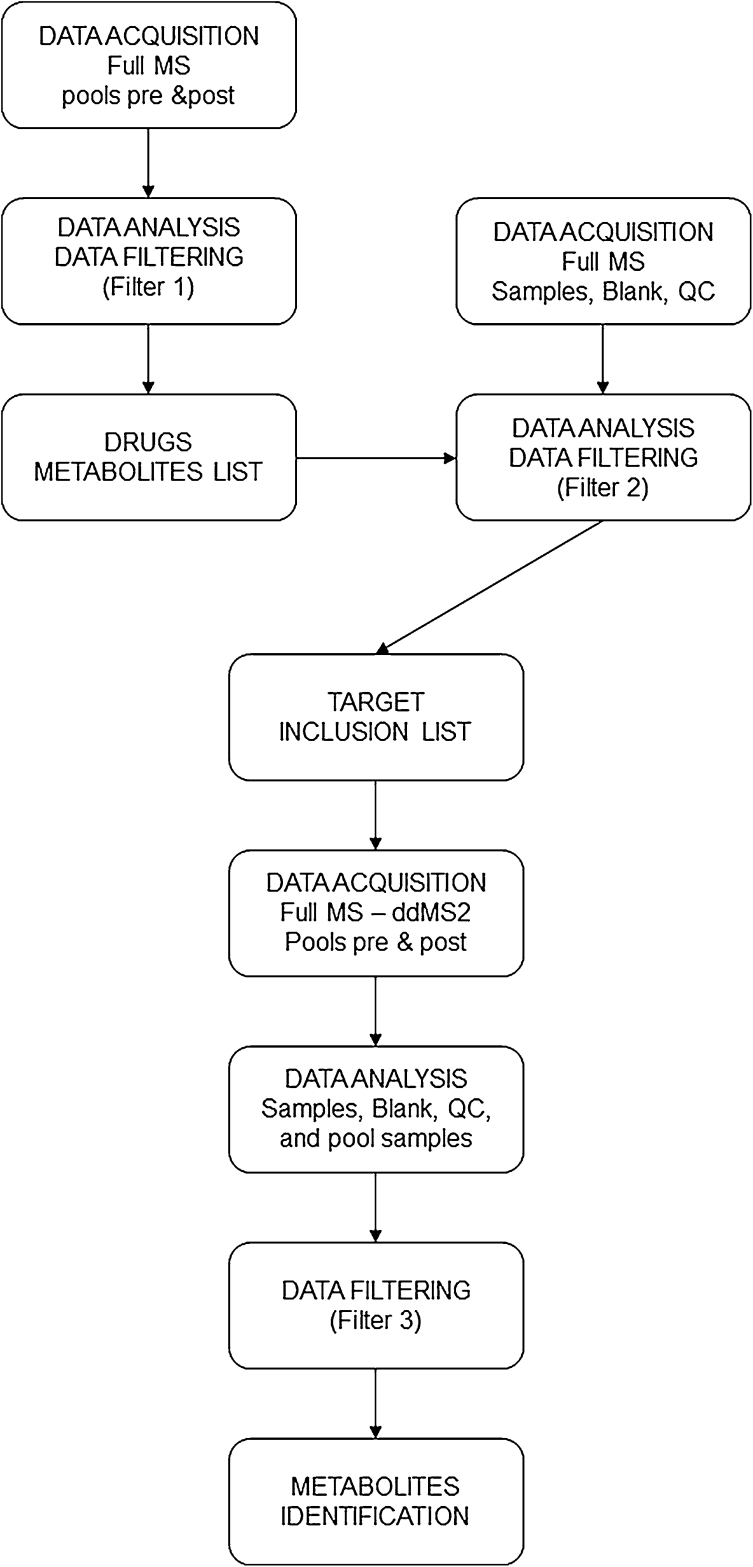
Data acquisition and data analysis workflow. The figure shows the different steps of data acquisition and data analysis pipeline. Filter 1: Area > 50,000; Retention time (RT) > 1 min. Filter 2: Group areas ≥ 50,000; p-value < 0.05; RT > 1 min; 0.2 ≤ ratio ≥ 5; no matches found in “exogenous drugs” mass list. Filter 3: Filter 2 plus at least one fragmentation spectra or one result from mzCloud database

A preliminary list of administered drugs (Table 1) and their endogenous metabolites was compiled using the “generate expected compound” node of the Compound Discoverer™ 2.1 (Thermo Fisher Scientific). The list includes parent compounds, administered drugs formula, and their possible transformation products. The “find expected compounds” node was used to search for compounds in the compound ions list provided by two “generate expected compound” nodes for every workflow. A more detailed illustration of this part of the workflow and its associated nodes is depicted in Fig. 1. Results were filtered for retention time (RT) greater than 1 min and groups area greater than or equal to 50,000 (Filter 1; Fig. 2). A mass list, called “exogenous drugs”, was created from the full-MS scans acquired from pre- and post-surgery pool samples.

**Table 1:** Administerd drugs

Administered drugs	Drug bank code
Heparin sodium	DBSALT000417
Protamine chloridrate	DB13700
Epinephrine	DB00668
Dopamine hydrochloride	DBSALT000508
Enoximone	DB04880
Fentanyl citrate	DBSALT000301
Phentolamine mesylate	DBSALT000980
Furosemide + Benzalkonium chloride	DB00695
Methylprednisolone hemisuccinate	DB14644
Midazolam hydrochloride	DBSALT000118
Milrinone lactate	DBSALT000891
Sodium nitroprusside	DBSALT000839
Alprostadil	DB00770
Sevoflurane	DB01236
Teicoplanin	DB06149
Thiopental sodium	DBSALT001409
Morphine hydrochloride	DBSALT001753
Cefuroxime sodium	DBSALT001160
Insulin	DB00030
Tranexamic acid	DB00302
Urapidil	DB12661
Charged drugs	
Cisatracurium besylate	DBSAT002671
Rocuronium bromide	DBSALT000575
Vecuronium bromide	DBSALT001200
Nitroglycerin	DB00727

List of administered drugs with their drug bank code

### Metabolomics workflow

The first experiment was conducted in Full MS mode. Procedural blank and 5 QC samples were injected in order to equilibrate the analytical platform. The QC were then injected at regular intervals (every 10 real samples) in order to check and correct the precision and reproducibility of individual mass spectral features (Dunn et al. 2011). Raw-files were processed by Compound Discoverer™ 2.1 using a processing workflow containing the “exogenous drugs” mass list in the “search mass list” node. Results were filtered for RT (greater than 1 min) groups area (greater than or equal to 50,000), groups area ratio (pre- vs post-surgery sample: ≤ 0.2 or ≥ 5.0) and groups ratio p value (less than 0.05) and screened against the “exogenous drugs” mass list (mass tolerance 3 ppm, RT tolerance 0.5 min) (Filter 2; Fig. 2). The m/z and RT values of each remaining compound were included in two target inclusion list (for each polarity) to be used in the second experiment to acquire MS^2^ information of selected metabolites present in the pre- and post-surgery pool samples.

Raw data files obtained in full-MS mode (samples, procedural blank, and QC) and data obtained in full-MS followed by data-dependent MS^2^ (pool pre and post) were processed. Pre and post pool samples were labeled as “identification only” samples which were used as a source for fragmentation data. The software does not report compounds for this sample type. Results were filtered for RT, groups area, *p* value, ratio and the presence of at least one fragmentation spectra (Filter 3; Fig. 2).

The identity of known compounds were verified based on HRMS, MS/MS fragmentation pattern and RT. The tentative identity of the discovered unknown compounds were assigned based on the interpretation of their CID-MS/MS (30 and 60 eV) fragmentation patterns. The workflow is depicted in Fig. 2.

### Data analysis

Raw data files were processed by Compound Discoverer™ 2.1 software for initial data processing, including peak detection, peak alignment and peak integration. Briefly, raw files were aligned with adaptive curve setting with 5 ppm mass tolerance and 0.4 min retention time shift. Unknown compounds were detected with a 5 ppm mass tolerance, 3 signal to noise ratio, 30% of relative intensity tolerance for isotope search, and 500,000 minimum peak intensity, and then grouped with 5 ppm mass and 0.2 min retention time tolerances. A procedural blank sample was used for background substraction and noise removal during the pre-processing step. Peaks with less than a 3-fold increase, compared to blank samples, and those detected in less than 50% of QCs and where the relative standard deviation (%RSD) of the QCs was greater than 30% were removed from the list. Peak areas, across all samples, were subsequently normalized to the total area of the corresponding samples to balance their differences in intensities that may have arisen from instrument instability. Metabolites identified in the processed raw data of mass spectral peaks were searched against both ChemSpider™ chemical structure database (3 ppm mass tolerance) and mzCloud spectral library (precursor and fragment mass tolerance, 10 ppm). Five data sources were selected via the ChemSpider database: Human Metabolome Database (HMDB), Kyoto Encyclopaedia of Genes and Genomes (KEGG), LipidMAPS, Biocyc, and Drugbank.

Reference standards were used to validate and confirm some significantly changed metabolites by comparing their MS/MS spectra and retention time.

### Statistical analysis

Clinical and surgical patient data were summarized using frequency and percentage for binary variables, whereas mean and standard deviation or median and range (min–max) were presented for continuous numeric variables (PASW Statistics 18.0, IBM Corp, Armonk, NY). Alterations in each compound were calculated using both a Student’s t test and a Student’s t-test with a Benjamini–Hochberg false discovery rate of 5% (Benjamini and Hochberg 1995). This yielded an adjusted *p* value for each metabolite termed *q* value. For each compound, groups area fold change was calculated and expressed as log2 scale (Compound Discoverer™ 2.1).

## Results

Fourteen newborns with TGA undergoing elective ASO were included in this study. Baseline and surgical characteristics are shown in Table 2. The mean age at surgery was 9 (3–15) days and pre-operative oxygen saturation was 86 ± 7. There were no significative differences in surgical characteristics among patients.

**Table 2:** Baseline characteristics and surgical characteristics

Baseline characteristics	TGA
N	14
Term n(%)	13 (93)
Male sex n (%)	11 (79)
Age at surgery (days)	9 (3-15)
Weight (Kg)	3.2 ± 0.4
SaO_2_ (%)	86 ± 7
Surgical characteristics	
Type of operation	ASO
Operation time (min)	230 ± 37
CPB time (min)	142 ± 28
Hypothermia time (min)	100 ± 21
Rewarming time (min)	36 ± 14
Minimum temperature (°C)	27.8 ± 2.2
Minimum temperature time (min)	64 ± 33

*TGA* transposition of great arteries, *SaO*_*2*_ arterial oxygen saturation, *CPB* cardiopulmonary bypass, *ASO* arterial switch operation

Values are expressed as frequency and percentage for binary variables, whereas mean and standard deviation or median and range (min–max) were presented for continuous numeric variables

All newborns received breast milk as minimal enteral feeding; feeding was discontinued at midnight before scheduled surgery in accordance with routine preoperative clinical practice. No major complications were observed after surgery.

### Univariate analysis

A mass list called “exogenous drugs” was compiled and includes 4392 drugs and drugs metabolite.

From the Full MS scan experiment 16,890 and 28,102 compounds with unique molecular weight and retention times were annotated from the positive and the negative modes respectively (Volcano Plot, Suppemental data Fig. S1). After data cleaninig to remove unreliable, inconsistent or unreproducible features (> 30%RSD^QC^) and background substraction, 9210 (ESI+) and 3081 (ESI−) compounds remained. Of these 11% (50%, ESI−) had RSD below 30%, 31% (28%, ESI-) below 20%, 29% (18%, ESI−) below 10% and, 29% (4%, ESI−) below 5%. Following data filtering 354 ESI(+) and 157 ESI(−) compounds remained and were subjected to a final visual inspection to eliminate compounds coming from in-source fragmentation of bigger molecules and metabolites deriving from naturally charged drugs (e.g. cisatracurium besylate, rocuronium bromide, vecuronium bromide and, nitroglycerin). In order to acquire MS^2^ information in the pre- and post-surgery pool samples m/z and RT values of each remaining compound were included in two target inclusion list (for each polarity). Raw data were added to the final analysis and the fragmentation scans were used only for the mzCloud search.

Raw data files were processed, and about 278 ESI+ and 93 ESI− metabolites remained after data filtering. Among the 371 discriminant metabolites (p < 0.05) this work led to the identification of 11 metabolites in human urine samples. The identification was either formally (when at least two physiochemical parameters, such as chromatographic retention time and MS/MS spectrum, matched those of our spectral library of reference compounds) or putatively (based on information from mzCloud and the interpretation of MS and MS/MS spectra), corresponding to levels 1 and 2 from the metabolomics standard initiative (Sumner et al. 2007) (Table 3).

**Table 3:** Putatively annotated and identified metabolites

	Ion description	Detected m/z	Predicted formula	Δmass ppm	RT (min)	RSD QC (%)	*P*	*q*	Ratio	Area fold change	ID level
N-Phenylacetylglutamine	[M + Na] + 1	287.09982	C13 H16 N2 O4	− 1.13	2.89	5	**0.004**	0.040	9.238	3.21	2
20 β-Dihydrocortisol	[M + H] + 1	365.23218	C21 H32 O5	0.20	5.31	4	**0.018**	0.098	8.150	3.03	2
Cortisol	[M + Na] + 1	385.19824	C21 H30 O5	0.57	10.05	7	**< 0.001**	0.007	0.170	− 2.55	2
Indole-3-carboxaldehyde	[M + H] + 1	146.06001	C9 H7 N O	0.25	5.28	4	**< 0.001**	0.001	0.018	− 5.83	2
Eicosatetraynoic acid	[M + H] + 1	297.18481	C20 H24 O2	0.15	4.74	11	**0.005**	0.051	0.072	− 3.79	2
trans-3-Indoleacrylic acid	[M + H] + 1	188.07047	C11 H9 N O2	0.72	5.28	4	**< 0.001**	< 0.001	0.002	− 9.04	2
Glycocholic acid	[M + Na] + 1	88.29858	C26 H43 N O6	0.23	14.61	5	**0.042**	0.150	10.866	3.44	1
Kynurenic acid	[M + H] + 1	190.04984	C10 H7 N O3	0.37	2.58	0.4	**0.004**	0.047	9.895	3.31	1
N-Acetyl-DL-tryptophan	[M + H] + 1 [2M − H] − 1	247.10748	C13 H14 N2 O3	1.10	5.28	3	**< 0.001**	< 0.001	0.001	− 10.73	1
		491.19403		0.05	5.28	27					
D-(+)-Tryptophan	[M + H − NH3] + 1	188.07047	C11 H12 N2 O2	0.67	5.28	1	**< 0.001**	< 0.001	0.001	− 10.35	1
5-hydroxy-L-tryptophan	[M + H] + 1	221.09193	C11 H12 N2 O3	0.42	1.25	0.4	**0.011**	0.076	8.856	3.15	1
Kynurenine	[M + H] + 1	209.09212	C10 H12 N2 O3	0.09	1.31	0.5	0.072	0.190	2.685	1.42	1
3-hydroxy-L-kynurenine	[M + H + MeOH] + 1	257.11285	C10 H12 N2 O4	1.42	1.07	2	**0.007**	0.062	4.450	2.15	2

Ratio = pre/post

Putatively annotated (Level 2) and identified metabolites (Level 1). Univariate analysis (*t*-test) between groups. Bold font indicates metabolites that have passed the defined level of significance (p < 0.05). Area fold change is expressed as log2 scale

Metabolites were distributed in 5 different pathways, including those regarding phenylalanine metabolism (N-phenylacetylglutamine), glucocorticoid byosynthesis (cortisol), tryptophan metabolism (indole-3-carboxaldehyde, indoleacrylic acid, kynurenic acid, tryptophan and 5-hydroxy-l-tryptophan), bile acid byosynthesis (glycocholic acid) and, omega-3 byosynthesis (eicosatetraynoic acid). Metabolites linked to the kynurenine pathway of tryptophan metabolism displayed the highest fold changes as high as 10.73 (log2) for tryptophan (Table 3).

Turning off the filters, we have found other two metabolites linked to the kynurenine pathway: kynurenine and 3-hydroxy-L-kynurenine (Table 3). N-Acetyl-tryptophan and 5-hydroxy-l-tryptophan were found both in positive and in negative polarity.

Using a critical q-value of 0.05, 7 metabolites were significantly altered even after post false discovery correction in the pre- vs post-surgery comparison.

## Discussion

Metabolomics is a relatively young branch of “omics” science and has been applied to several studies in critical care and neonatal medicine (Fanos et al. 2013). However, previous untargeted metabolomic approach have ignored confounders such as drugs and feeding. Compounds deriving from the analysis of biological samples collected from critically ill infants are known to be contaminated by compounds deriving from treatments such as drugs and parenteral or enteral nutrition.

In this study the metabolic variation between pre- and post-surgery urine samples collected from TGA patients was investigated with high performance liquid chromatography coupled to FTMS for non-polar metabolites. Before and, above all, during cardiac surgery, several drugs are administered to our study newborns therefore the primary objective of this study was to develop a robust method to clear metabolites deriving from these drugs, out of the urinary metabolites profile. Our analytical approach enabled us to uncover the changes in urine composition strongly correlated with the surgery. To the best of our knowledge, this is the first time that a list of drugs, usually administered to critically ill infants, is compiled for this purpose. We believe that this novel approach will be extremely useful for the correct identification of endogenous metabolites triggered by an acute event, such as cardiac surgery in infants with CHD.

Metabolomic studies in infants with CHD undergoing CPB are scarce and they are designed to selectively extract information regarding a group of related metabolites from a complex mixture of biomolecules present in biological samples. Recently Correia et al. (2015) reported substantial differences in metabolic profile in pre- and post-operative plasma samples of patients with different types of CHD, moreover a significant difference in the preoperative metabolic fingerprints of neonates compared to older infants has been also observed (Davidson et al. 2018). Based on these results, we decided to use a homogeneous population, like TGA newborns, for our metabolomic study in order to overcome differences in metabolic profile due to age, surgery and/or disease severity, and to focus on CPB metabolic response only.

### Interpretation of results

**Fig. 3:**
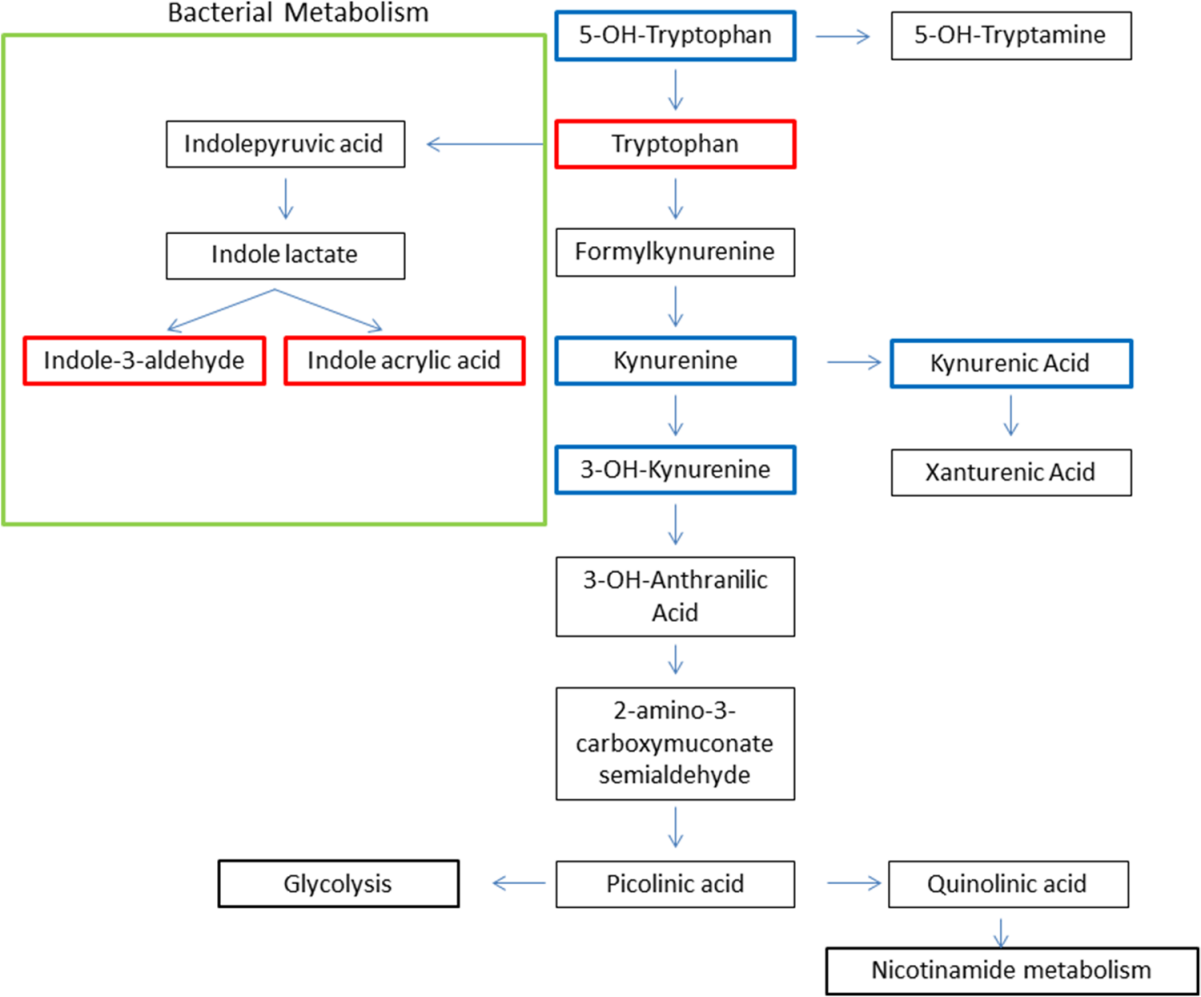
Alterations in tryptophan metabolism. About 90% of the tryptophan (TRP) is metabolized along the kynurenine (KYN) pathway (KP), 3% is metabolized into serotonin (5-OH-tryptamine) while, the rest is degraded by the gut microbiota to produce indole and its derivatives. In neuroinflammatory condition, the KP pathway is up-regulated leading to the production of several neuroactive metabolites. Peripheral KYN and TRP can be transported across the blood brain barrier and be metabolized into kynurenic acid (KYNA), by astrocytes, or into quinolinic acid (QUIN) by microglia. KYNA has anti-inflammatory, immunosuppressive and antioxidant functions. QUIN can lead to neuronal dysfunction and/or death whereas picolinic acid is neuroprotective and is an efficient metal chelator. Indole-3-aldehyde and indole acrylic acid are produced by gut bacterial metabolism of TRP and can modulate CNS inflammation. TRP metabolites marked in bold have been found to be altered in the current study. Different colors indicate an increases (red) or decreases (blue) in metabolite area in the post- vs pre-surgery sample, (KEGG map 00380) (Color figure online)

We found that urine metabolites profile of TGA patients changed after the surgery, in a direction that suggests a marked alteration in peripheral tryptophan (TRP) metabolism (Fig. 3) via the kynurenine pathway (KP). The KP pathway is initiated by the conversion of TRP to N-formylkynurenine by any of these enzymes: indoleamine 2,3-dioxygenase 1 (IDO1), IDO2, or tryptophan 2,3-dioxygenase (TDO). The resulting N-formylkynurenine is further degraded to kynurenine (KYN), which is a precursor of bioactive compounds, including quinolinic acid (QUIN), kinurenic acid (KYNA), picolinic acid, and 3-hydroxyanthranilic acid. In mammals, the KP has been reported to account for over 90% of peripheral TRP catabolism (Leklem 1971) and plays a crucial role in the regulation of the immune response in the context of inflammation (Wirthgen and Hoeflich 2015). In the brain the various KP product can have either neurotoxic, neuroprotective or immunomodulatory effects (Vecsei et al. 2013). Among them, KYNA, mostly produced by astrocytes (Guillemin et al. 2001), is an antagonist of all ionotropic glutamate receptors and thus can potentially block some of the effects of the excitotoxin QUIN and of other neurotoxins. Furthermore, KYNA is an antioxidant capable of scaveging free radicals (Lugo-Huitron et al. 2011).

Our findings exhibit an increase in TRP, trans-3-indoleacrylic acid, indole-3-carboxaldehyde whereas 5-hydroxy-l-tryptophan, KYN, 3-hydroxy-KYN, and KYNA are decreased in the post-surgery urine samples. Interestingly, this dysregulation of the KP homeostasis also manifests in the blood of patients with neuroinflammatory diseases. KYNA was found to be decreased in the blood of patients suffering from glioblastoma (Adams et al. 2014) and in patients with Alzheimer’s and Parkinson’s diseases (Chatterjee et al. 2018; Lim et al. 2017) compared to healthy subjects. In addition to lower plasma level of KYNA, increased levels of KYN and KYNA were detected in glioblastoma cells and in the CSF of patients with Alzheimer’s disease where KYNA amount correlated with the expression of P-tau and the soluble intercellular adhesion molecule-1 which are biomarkers of inflammation (Opitz et al. 2011).

Elevated plasma or urine level of TRP along with lower peripheral level of KYNA and KYN in post- versus pre-surgery samples might reflect an increased synthesis and transport of KYN through the blood–brain barrier as a substrate for local synthesis of KYNA, knowing that KYNA is hardly able to cross the blood–brain barrier (Fukui et al. 1991). Thus, what is happening in the periphery may not represent what happens in the brain.

Open-heart surgery with application of CPB is known to be associated with acute activation of cellular and hormonal factors leading to postoperative stress response, clinically termed the systemic inflammatory response syndrome (SIDS). The CPB-generated inflammatory/stress response has been well demonstrated, and the younger the age of the child, the more robust is the response (Alcaraz et al. 2005). Cortisol is one of the most important stress hormones and its secretion is proportional and positively correlated to the severity of surgical stimuli (Bangalore et al. 2019). In our study group even in presence of pre-CPB glucocorticoid administration, we found a higher level of cortisol in post-surgery urine with respect to pre-surgery samples (Table 3) (Crow et al. 2014). Cortisol activates TDO and increases KYN production, which can be used as KYNA precursor (Niimi et al. 1983).

CPB carries significant risks to the brain. Neurodevelopmental impairment is emerging as one of the most important current challenges for survivors after pediatric cardiac surgery. Cerebral injury from cardiac surgery is primarily ischemic, secondary to embolism and/or cerebral hypoperfusion (Hogue et al. 2006). Neuronal injury is likely exacerbated by inflammatory processes resulting from CPB and ischemia/reperfusion (Laffey et al. 2002). The central nervous system has its own structures responsible for the neuroinflammation response, being microglia and reactive astrocytes the principal actors throughout the production of pro-inflammatory agents such as glutamate, TNF-α, prostaglandins, IL-6 and, reactive oxygen species (ROS) (Niranjan 2014). IDO 1 and IDO 2 are upregulated by inflammatory stimuli such as IFN-ϒ, TNF-α and lipopolysaccharides (Meyer et al. 1992), these two enzymes activates the KP pathway.

From all these data, we can suppose that during the CPB period, there is a rise in stress hormones (e.g. cortisol) and in inflammatory cytokines both in the periphery and in the brain. The subsequent induction of both TDO and IDO resulted in KP pathway activation with an increase in KYN production. Given the permeability of the blood brain barrier to KYN (Fukui et al. 1991) it can be taken up and metabolized within the CNS into KYNA by astrocytes or into QUIN by microglia. Under physiological conditions KYNA is produced from KYN by kynurenine aminotransferases (KATs). Alternative routes for KYNA synthesis in presence of ROS has been described. KYN can be converted to KYNA in presence of hydrogen peroxide or KYNA formation can result from reaction of KYN or indole-3-pyruvic acid, deriving from deamination of TRP, under conditions generating free radicals (Ramos-Chavez et al. 2018).

We also identified two metabolites, indole-3-carboxaldehyde and indoleacrylic acid, which are are exclusively produced by gut bacterial metabolism of TRP (Gao et al. 2018). Beneficial effect of indoleacrylic acid has been proposed, hinged on its ability to promote intestinal epithelial barrier function and mitigates inflammatory responses (Wlodarska et al. 2017). Indole-3-carboxaldehyde is a biologically active metabolite which act as a receptor agonist at the aryl hydrocarbon receptor in intestinal immune cells, stimulating the production of IL-22 which facilitates mucosal reactivity (Zelante et al. 2013). Furthermore, has been recently demonstrated that the metabolites of dietary TRP produced by the commensal flora modulate microglia-astrocyte interactions and CNS inflammation (Rothhammer et al. 2018).

Among the identified metabolites, other 2 were part of the KP pathway, 5-hydroxy-l-tryptophan and 3-hydroxy-L-kynurenine which were higher in the pre- with respect to the post-surgery sample. Tryptophan is an essential precursor for KP pathway as well for serotonin/melatonin pathway. During acute or chronic inflammation, TRP is preferentially catabolized through the KP route rather than serotonin/melatonin pathway resulting in a decrease of serotonin production. This was supported by a decrease, in our post-surgery sample, of 5-hydroxy-l-tryptophan that give rise to serotonin and melatonin.

Kynurenine-3-monooxygenase converts KYN into the neurotoxic free-radical generator, 3-hydroxy-L-kynurenine, which can be taken up by kynurenine aminotransferase to produce xanthurenic acid. 3-hydroxy-L-kynurenine is lower in the post-surgery samples indicating a down-regulation of this branch with a preferential transport of KYN to the brain for KYNA synthesis.

### Strenghts and limitations of the study

To our knowledge, this work is the first metabolomic study in TGA newborns undergoing surgery for CHD. This study demonstrates that we can accurately clear out metabolites deriving from administered drugs, and that changes occur in the metabolic profile as response to the surgical insult.

This study has weakness and strenght. The main weakness is that our pilot study is limited by its small sample size and larger studies are needed to validate our results. Moreover, we are aware that we can correct for the drug metabolites but nonetheless it can not be ruled out the effect of the administered drugs on the overall metabolism. However, a significant strenght of the study was the data analysis workflow built to overcome the confounding factors, this makes possible to reliably measure the changes of markers of interest.

### Clinical implications and future directions

The untargeted approach used in this study showed the power to explore metabolites that differentiate between pre- and post-surgery samples. The differentiating metabolites found in this work have guided our focus to the kynurenine pathway. With confidence we can now progress these metabolites to a quantitative targeted metabolomic analysis in a larger cohort of CHD patients. The amount of these metabolites will be correlated to our gold standard of impaired neurodevelopment, GFAP, and with data obtained from post-surgery neurological and neurodevelopmental test. This may provide the robust biomarker panel needed for prediction of brain damage. Early identification of patients at risk for adverse neurodevelopmental outcomes is of paramount importance for both preemptive interventions and actuation of assisted neurodevelopment programs.

## Electronic supplementary material

Below is the link to the electronic supplementary material.

Supplementary material 1 (XLSX 8408 kb)

Supplementary material 2 (TIFF 295 kb). **Fig. S1** Volcano plots for the pre vs. post-surgery samples. Volcano plot between pre- and post-surgery samples in positive (ESI+) and negative (ESI−) mode. Fold change (log2) on X-axis plotted against p-value (− log10) on Y-axis. The horizontal line marks the p = 0.05 and the vertical lines mark a fold change of ±1.0. Compared with the pre-surgery sample, all plots in the upper red quadrant indicates a significantly upregulated metabolite in the post-surgery sample, whereas all plots in the left upper quadrant indicates the opposite

Supplementary material 3 (XLSX 131 kb)
