# Supplementary figures and images for: Urinary metabolomics reveals kynurenine pathway perturbation in newborns with transposition of great arteries after surgical repair
Source: Metabolomics. 2019 Oct 28;15(11):145. doi: 10.1007/s11306-019-1605-3 (PMC6817811; doi:10.1007/s11306-019-1605-3)

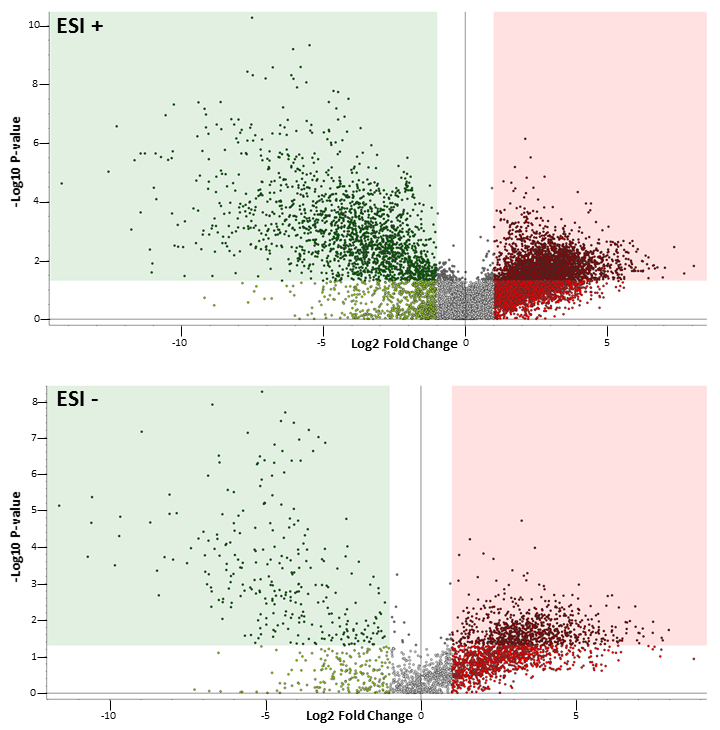

Supplement: Supplementary file 2 — Supplementary material 2 (TIFF 295 kb). Fig. S1 Volcano plots for the pre vs. post-surgery samples. Volcano plot between pre- and post-surgery samples in positive (ESI+) and negative (ESI−) mode. Fold change (log2) on X-axis plotted against p-value (− log10) on Y-axis. The horizontal line marks the p = 0.05 and the vertical lines mark a fold change of ±1.0. Compared with the pre-surgery sample, all plots in the upper red quadrant indicates a significantly upregulated metabolite in the post-surgery sample, whereas all plots in the left upper quadrant indicates the opposite [file 11306_2019_1605_MOESM2_ESM.tif]
